# Heroin overdose masquerades as methomyl poisoning: a case report

**DOI:** 10.1186/s13256-019-2234-1

**Published:** 2019-11-05

**Authors:** Yutti Amornlertwatana, Paitoon Narongchai, Siripun Narongchai

**Affiliations:** Department of Forensic Medicine, Faculty of Medicine, Chiang Mai University, Chiang Mai, 50200 Thailand

**Keywords:** Suicide, Homicide, Accident, Methomyl, Heroin, Insecticide, Environmental Protection Agency (EPA), Insurance

## Abstract

**Background:**

Methomyl is the most common cause of suicidal death but heroin is the most common cause of accidental death. The problem is to determine the exact cause and manner of death between methomyl or heroin toxicity. The evidence from autopsy includes crime scene investigation, toxicological analysis by liquid chromatography with mass spectrometry, and knowledge of methomyl and heroin intoxication.

**Case presentation:**

A 35-year-old Thai man and a 30-year-old Thai woman were found showing evidence of cyanosis, with a fine froth around the nose and mouth. Postmortem interval time was 24 hours. According to the police’s and hotel owner’s records, the couple stayed together for 1 day before being found dead in bed, naked, with a foul and a fine froth around the nose and mouth. A methomyl insecticide sachet and a plastic box containing white powder form of heroin were found at the scene. Laboratory tests of the male corpse identified the presence of methomyl in the blood of the stomach and morphine, codeine, methadone, and tramadol in the systemic blood. Blood cholinesterase enzyme activity and morphine concentration was 3416 U/L or 53% (normal 6400 U/L) and 0.058 μg/ml respectively. Laboratory test of the female corpse identified the presence of methomyl in the stomach and blood, and cholinesterase enzyme activity was 1965 U/L or 30.7%.

**Conclusions:**

Cause of death of the male corpse was deemed to be due to heroin intoxication as the blood concentration of morphine was more than the lethal concentration with a morphine/codeine ratio of more than 1:1. Methomyl intoxication of the male corpse was unlikely to be the cause of death because methomyl systemic blood concentration was found to be very low, < 2.5 μg/ml, and cholinesterase enzyme levels did not indicate lethal activity (< 10–15% of normal). The main problem regarding an insurance claim is that the policy will not pay out in the case of heroin-associated deaths, as it is an addictive drug. The policy would pay out on death by suicide with methomyl insecticide, which was not prohibited by the insurance company after 1 year of insurance. So, it is not clear whether or not the family will receive money from the insurance company.

## Introduction

Insecticides are widely used in agriculture in Thailand. The common insecticides are organophosphorus (OP) compounds and carbamates (CM), which act as contact poison to insects without being phytotoxic to the plant. These poisons are toxic to humans and every year more than 200,000 people die due to these poisons globally (World Health Organization). Methomyl is classified as a Restricted Use Pesticide (RUP) by the US Environmental Protection Agency [1, 2]. Methomyl was analyzed and available for purchase in 1966 as an effective spectrum insecticide [3]. In humans, poisoning is due to inhibition of the action of cholinesterase enzymes at the neuromuscular junctions in the nervous system and at the muscarinic receptors [4]. A reduced cholinesterase enzyme level in blood is indicative of poisoning due to these insecticides [5]. Methomyl is a CM that with human exposure results in respiratory failure due to breathing arrest, paralysis of respiratory muscles, and airway obstruction by hypersecretion [6]. In Thailand, methomyl is marketed under the trade name of Lannate® 40%SP. Thailand is one of the most popular destinations for international tourists. It is famous for its temples, sea beaches, nightlife, and prostitution. As tourists come to the country for merrymaking, they are often exposed to narcotic drugs by drug traffickers. Despite stringent laws, an illegal drugs and substance trade has flourished in the country.

Heroin intoxication and death is the most common cause of accidental death in Thailand. The histopathology associated with death by heroin and other opioids is cyanosis and pulmonary edema, which is due to suppression of the respiratory center in the brainstem and respiratory failure [7]. Its histopathology is likely to be similar to methomyl intoxication. Heroin is rapidly metabolized to 6-monoacetylmorphine (6-MAM), morphine, and codeine [7]. The ratio of morphine/codeine metabolites of heroin is more than 1 [8]. Morphine is the principle metabolite of heroin and the blood concentration of morphine is used to determine that heroin is the cause of death [8]. Laboratory evidence of fatality which indicates heroin intoxication can be determined by a free morphine blood concentration of 0–2800 ng/ml. The ratio of free to total morphine is more than 0.290 [9]. The median blood concentration of morphine in overdose deaths is 0.25 mg/L or 0.25 μg/ml and is within statistically acceptable limits of the concentrations recorded in accidental deaths of heroin users (0.23 mg/L or 0.23 μg/ml) [10].

In the insurance company policies of Thailand, if a patient dies within a year of taking out a policy, then money cannot be claimed from the insurance company. However, if a person dies by committing suicide as a result of methomyl poisoning more than a year after taking out a policy, then the insurance company will have to pay out. However, if a person dies as a result of heroin intoxication, then money cannot be claimed from the insurance company.

We report a case in which a 35-year-old Thai man suspected of suicidal CM poisoning was a victim of heroin overdose.

## Case presentation

A 35-year-old Thai man and his mistress, a 30-year-old Thai woman, were found dead in a hotel room in Chiang Mai, Thailand. The postmortem time of death was approximately 24 hours. The crime scene had a plastic box which contained some white powder and a container with a label that read Lannate® 40%SP also in the form of white powder which was methomyl insecticide. Both bodies were subjected to postmortem examination. During an autopsy at the Department of Forensic Medicine in Chiang Mai University, a copious amount of fine froth came out of their mouths and nostrils. There was bluish discoloration of their faces which was suggestive of cyanosis. No external injuries were noted. On opening the stomachs, a peculiar smell came from the greenish-brown gastric contents of both corpses. The mucosa of the stomach walls showed general submucosal hemorrhagic gastritis. The brains were edematous. The respiratory passages and the lungs were filled with fine froth. The liver and kidneys of both corpses showed features of congestion. The body fluids and the white powder recovered from the crime scene were subjected to chemical analysis. The viscera were sent for histopathological examination.

Analysis by liquid chromatography with tandem mass spectrometry (LC-MS-MS) was done: femoral arterial blood, 100 ml; urine, 100 ml; gastric contents, 100 ml. The laboratory reports of the Thai man and woman are presented in Table 1.

**Table 1:** The substances and laboratory results

Sample	Laboratory results			
	substance	Result of male/female corpses	Method	Sensitivity
1. Heart blood	1. Morphine	0.058/negative μg/ml	LC-MS-MS	Morphine: Codeine ≥ 5.8
	2. Codeine	< 0.010/negative μg/ml	LC-MS-MS	
	3. Methamphetamine	Negative/negative	LC-MS-MS	
	4. Benzodiazepines	Negative/negative	LC-MS-MS	
	5. Tramadol	0.66/negative μg/ml	LC-MS-MS	
	6. Methadone	0.071/negative μg/ml	LC-MS-MS	
	7. EDDP	0.010/negative μg/ml	LC-MS-MS	
	8. Methomyl	< 2.5/6.8 μg/ml	LC-MS-MS	
	9. ChE activity	3416/1965 U/L	Spectrophotometry	6400–8200
2. Femoral blood	1. Alcohol	Negative/negative	GC-MS	
3. Gastric contents	1. Methomyl	Positive/positive	TLC	
	2. ChE inhibitor	Positive/positive	Spectrophotometry	
4. Containers	1. Methomyl	Positive	TLC	
	2. Heroin	Positive	TLC	

*ChE* cholinesterase, *EDDP* 2-ethylidene-1,5-dimethyl-3,3-diphenylpyrrolidine, *GC-MS* gas chromatography-mass spectrometry, *LC-MS-MS* liquid chromatography with tandem mass spectrometry, *TLC* thin-layer chromatography

## Materials and methods

### Sample conditions

This study involves a male corpse who died with his mistress at a hotel in Chiang Mai, Thailand. Methomyl intoxication was initially suspected. The autopsy was performed out at the Department of Forensic Medicine, Faculty of Medicine, Chiang Mai University, Chiang Mai, Thailand. The study was approved by the Department for the Civil Law of Thailand. The methomyl, trade name Lannate® 40%SP, was purchased from the retailer DuPont Co., Ltd., Thailand.

### Experimental design

A complete autopsy was performed at Department of Forensic Medicine, Chiang Mai University, Thailand. Sample specimens including blood and stomach contents, and witness materials were collected at the scene. At the mortuary, the lungs, liver, heart, and kidneys were removed and histological analysis completed. Paraffin-embedded sections stained with hematoxylin and eosin were examined using light microscope.

### Laboratory analysis

The autopsy on a 35-year-old Thai male corpse and a 30-year-old Thai female corpse, estimated time after death was 24 hours, reported no evidence of external injury. The autopsy was carried out using the following:

1. Blood, urine, and gastric contents were analyzed using liquid LC-MS-MS to assess the presence of the substances: methomyl insecticide, heroin, 6-MAM, morphine, and others.
2. The system used was an Acquisition Method Report with Agilent Technologies 6460 Triple Quad LC/MS with Column of Eclipse Plus C18 1.8 mm 2.1 × 100 mm. The specimens (that is, femoral blood 100 ml, urine 100 ml, and gastric contents 100 ml) were collected from the corpses and analyzed immediately by LC-MS-MS.
3. Method Path was http://massbank.jp/Search

## Results

**Fig. 1:**
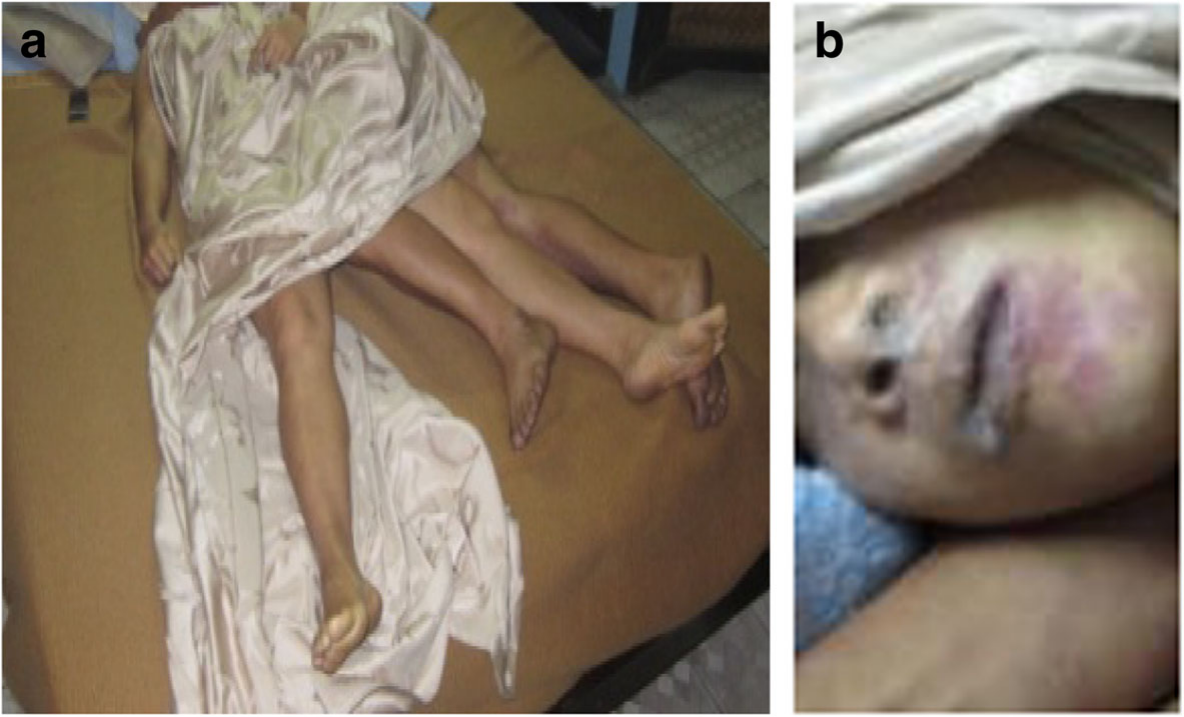
**a** The scene of investigation included 2 intertwined corpses showing evidence of cyanosis, **b** The corpses showed evidence of cyanosis, with a fine froth in the mouths and noses

**At the scene of investigation** two corpses were present in the rented room. They both had large amounts of fine froth in the nose and mouth and cyanosis was evident (Figs. 1a, 1b). They were known to have problems in their personal life as he had a wife and he could not stay with his lover. Trace evidence was collected from the scene, including heroin in the form of white powder in a plastic box and a container with methomyl insecticide in the form of white powder. The odor of hydrogen sulfide was present in the room emanating from the stomachs of the victims.

**Fig. 2:**
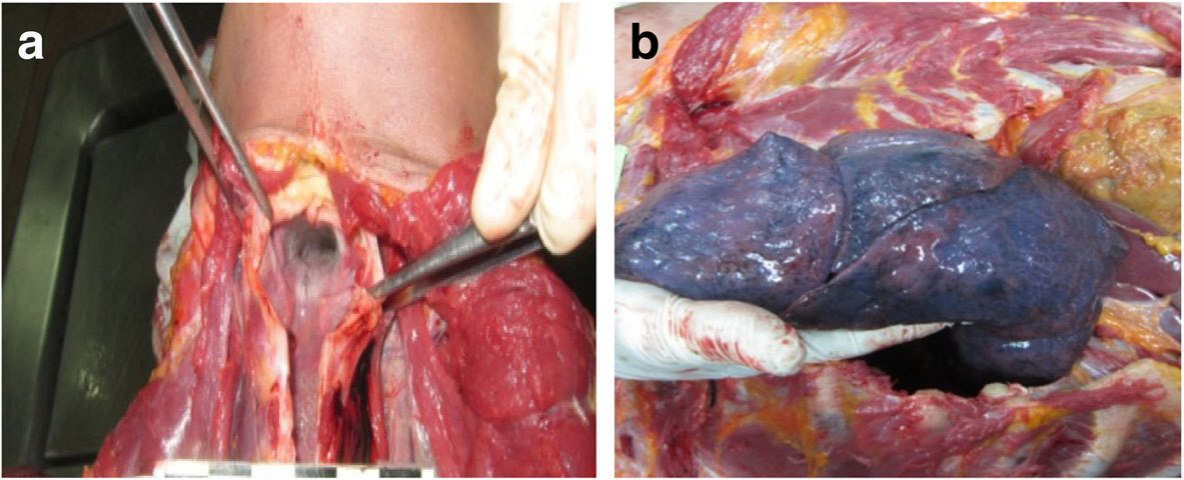
**a** Pulmonary congestion and edema **b** with fine froth in the airway passages

**Fig. 3:**
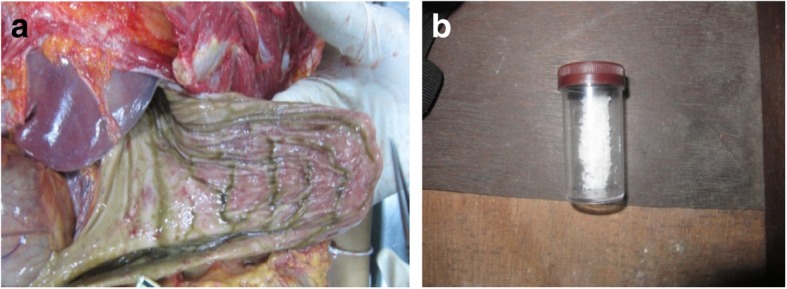
**a** General submucosal hemorrhagic gastritis and heroin in the form of a white powder, **b** contained in a plastic box collected at the scene

**At the autopsy room** and microscopic pathology of both corpses. The pathological findings were edema of the brain and lungs, fine froth in the airway passages, congestion of the liver and kidneys, and general submucosal hemorrhagic gastritis with hydrogen sulfide odor and green-brown color of gastric contents (Figs. 2a, 2b and 3a). The white powder retrieved from the scene of investigation was analyzed by LC-MS-MS. The laboratory results showed heroin in the plastic box (Fig. 3b), its metabolites in the male body, and methomyl insecticide in the stomachs and blood of the body, and the container (Table 1).

**At the laboratory:** The results of laboratory investigation are shown in Table 1.

## Discussion and conclusion

The cause of death of the case of the Thai man was opiate/opioid intoxication, most likely due to heroin more than methomyl. The blood concentration of morphine was 0.058 μg/ml, which is a lethal concentration, and the ratio of morphine/codeine was 5.8 times more than 1:1. However, methomyl insecticide was not the principal cause of death because the blood concentration of methomyl was < 2.5 μg/ml, which is less than the lowest lethal blood concentration ever reported (> 3.24 μg/ml) but blood concentration of the female corpse was 6.8 μg/ml. In addition, the cholinesterase enzyme activity of male corpse did not decrease by more than 90% which would be the case in death by methomyl, but in this case was only 53%. Methomyl may be a concomitant cause of death in this case but was not the major cause. It is the policy of insurance companies of Thailand that if a patient dies within a year of taking out a policy, then the legal situation as to whether the patient committed suicide using methomyl or by intoxication with heroin, or any opioids that are narcotic drugs, is irrelevant. The money cannot be claimed from the insurance company under this condition. However, if a patient committed suicide as a result of methomyl poisoning more than a year after taking out the policy, then it is relevant and the insurance company will have to pay out, but if the patient died as a result of heroin intoxication the money cannot be claimed from the insurance company. In this case we concluded that heroin intoxication was the cause of death, not methomyl insecticide. However, if thorough laboratory techniques had not been used in this case, the cause of death would have been methomyl insecticide intoxication because of the evidence found at scene of death. In addition, he died with his mistress which is common in Thailand. This is one of the principal problems faced by forensic medicine teams in Thailand because there are limitations to death investigation techniques in many areas. Forensic pathologists may be judged by the justice system if they give an incorrect diagnosis or opinion in distinguishing between deaths as a result of methomyl or heroin intoxication. The conclusion of the case of the Thai man was a suicide and the possibility of heroin intoxication was based on the scientific data of the laboratory. Insurance cannot pay out the money because it is against the policy and agreement before acceptation.

## Data Availability

All kinds of data of this case or all cases of unnatural cause of death are kept as secret processes because all unnatural deaths must be processed by criminal law.
